# Mechanism of Kch activation by Zn^2+^

**DOI:** 10.1093/pnasnexus/pgag284

**Published:** 2026-08-31

**Authors:** Brian Morote-Costas, Yaping Pan, Lie Wang, Xiaochen Bai, Steve W Lockless, Ming Zhou

**Affiliations:** The Verna and Marrs McLean Department of Biochemistry and Molecular Pharmacology, Baylor College of Medicine, Houston, TX 77030, USA; The Verna and Marrs McLean Department of Biochemistry and Molecular Pharmacology, Baylor College of Medicine, Houston, TX 77030, USA; The Verna and Marrs McLean Department of Biochemistry and Molecular Pharmacology, Baylor College of Medicine, Houston, TX 77030, USA; Department of Biophysics, University of Texas Southwestern Medical Center, Dallas, TX 75390, USA; Department of Biology, Texas A&M University, College Station, TX 77843, USA; The Verna and Marrs McLean Department of Biochemistry and Molecular Pharmacology, Baylor College of Medicine, Houston, TX 77030, USA

**Keywords:** bacterial K^+^ channel, cryo-EM, K^+^ channel gating, RCK domains

## Abstract

*Escherichia coli* has a single K^+^ channel gene, *kch*, that produces a K^+^ channel with six transmembrane segments and a cytosolic Regulate Conductance of K^+^ channels (RCK) domain. RCK domains are present in both eukaryotic and prokaryotic organisms and known to regulate ion channel activity upon binding of an ion or a nucleotide, however, how Kch is regulated remains unknown. Here, we show that Zn^2+^ or Cu^2+^ activates Kch via the RCK domain. Structural studies reveal that eight RCK domains assemble into a gating ring that can assume three conformations, closed, intermediate, and open, and that Zn^2+^ promotes the open conformation by stabilizing the assembly interface. These results expand our knowledge on Kch and on RCK-mediated regulation of ion channels.

Significance Statement Kch is the only K^+^ channel in *Escherichia coli*, and based on its sequence, Kch is a ligand-gated channel with a gating ring on the cytosolic side. However, neither the activating ligand nor the mechanism of gating is resolved. This article reports the discovery of Zn^2+^ and Cu^2+^ as agonists for Kch, and structures of the gating ring in three conformations, open, intermediate, and closed. These results enhance our knowledge of Kch and will facilitate understanding of Kch function in *E. coli*.

## Introduction

The *kch* gene encodes the only K^+^ channel in *Escherichia coli* genome (1, 2). The precise physiological role of Kch in *E. coli* remains elusive, although studies have shown that in multicellular bacterial communities such as *Bacillus subtilis* biofilms, K^+^ channels could facilitate intra and inter species electrical signaling regarding metabolic competition, predation, and a variety of stresses affecting the biofilm (3, 4). Deletion of the *kch* gene does not produce a significant phenotype under common growth conditions; however, overexpression of Kch is toxic to *E. coli* (5). Kch is thought to alter membrane potentials in response to changes in cellular metabolic conditions or stresses, but it is not clear which metabolic signal triggers a response (3, 5). Recent work has linked Kch activity to porin permeability and, consequently, to antibiotic susceptibility in *E. coli*, with *kch* knockout strains exhibiting increased resistance to porin-dependent antibiotics (6).

The *kch* gene encodes a polypeptide of 417 amino acids, which is predicted to have a membrane-embedded domain composed of a canonical K^+^ channel of six transmembrane segments (TM), and a C-terminal cytosolic Regulate Conductance of K^+^ channels (RCK) regulatory domain (7). Studies have shown that in addition to the full-length Kch protein, an alternative start codon, methionine 240, allows for translation of an isolated RCK domain (residues 240–417) (1, 8) (Fig. S1a). A standalone RCK domain assembles with a full-length Kch, and four such assemblies form a homotetramer, in which there is a tetrameric 6TM K^+^ channel pore and an octameric RCK gating ring. This configuration has been observed in previous studies of Kch (9–11) (Fig. S1b). Similar octameric assembly of RCK domains are observed in other RCK regulated channels (12–15).

The structure of an isolated RCK domain from Kch was determined previously by X-ray crystallography, revealing a homodimer with each RCK domain having the α/β fold (Rossmann-like nucleotide-binding fold) and a dimer interface formed by a helix–loop–helix motif (HLH) from the C-terminus of each domain (8). Similar to RCK domains in other K^+^ channels or K^+^ transport systems such as MthK, TrkHA, and KtrAB, this dimer is the basic building block for higher-order assemblies (13, 16–19). Four RCK dimers form a ring-like structure, the gating ring, at the intracellular side of the channel (10). RCK domains are known to bind nucleotides such as ATP or NADH (19–21) or to ions such as Ca^2+^ or Na^+^ (13, 16, 22–24) and give their associated channels the ability to sense intracellular signals that regulate channel gating. The RCK gating rings exert their regulatory effect by undergoing ligand-induced conformational changes (24–26). Interestingly, the RCK domain in Kch does not have the consensus GXGXXG nucleotide-binding motif or recognizable cation binding motifs like the calcium bowl (14, 27). There have been attempts at determining the full-length structure of Kch, which yielded low resolution projection structures that provided evidence for the presence of an RCK gating ring, but lacked details (9, 10, 28). Thus, the goal of the current research is to identify activating ligands for Kch and determine the mechanism by which the RCK gating ring can modulate channel activity.

To better understand the mechanism governing Kch regulation, we expressed, purified, and reconstituted Kch into liposomes for functional characterization, and we identified Zn^2+^ and Cu^2+^ as agonists of Kch. We then determined the structures of the octameric RCK gating ring in three different conformations that highlight significant conformational changes associated with gating, and defined a potential binding site for the metal ions. These findings are an important step toward understanding of Kch function in *E. coli* and underscore the general principles of RCK-mediated K^+^ channel regulation.

## Results

### Activation of Kch

**Figure 1:**
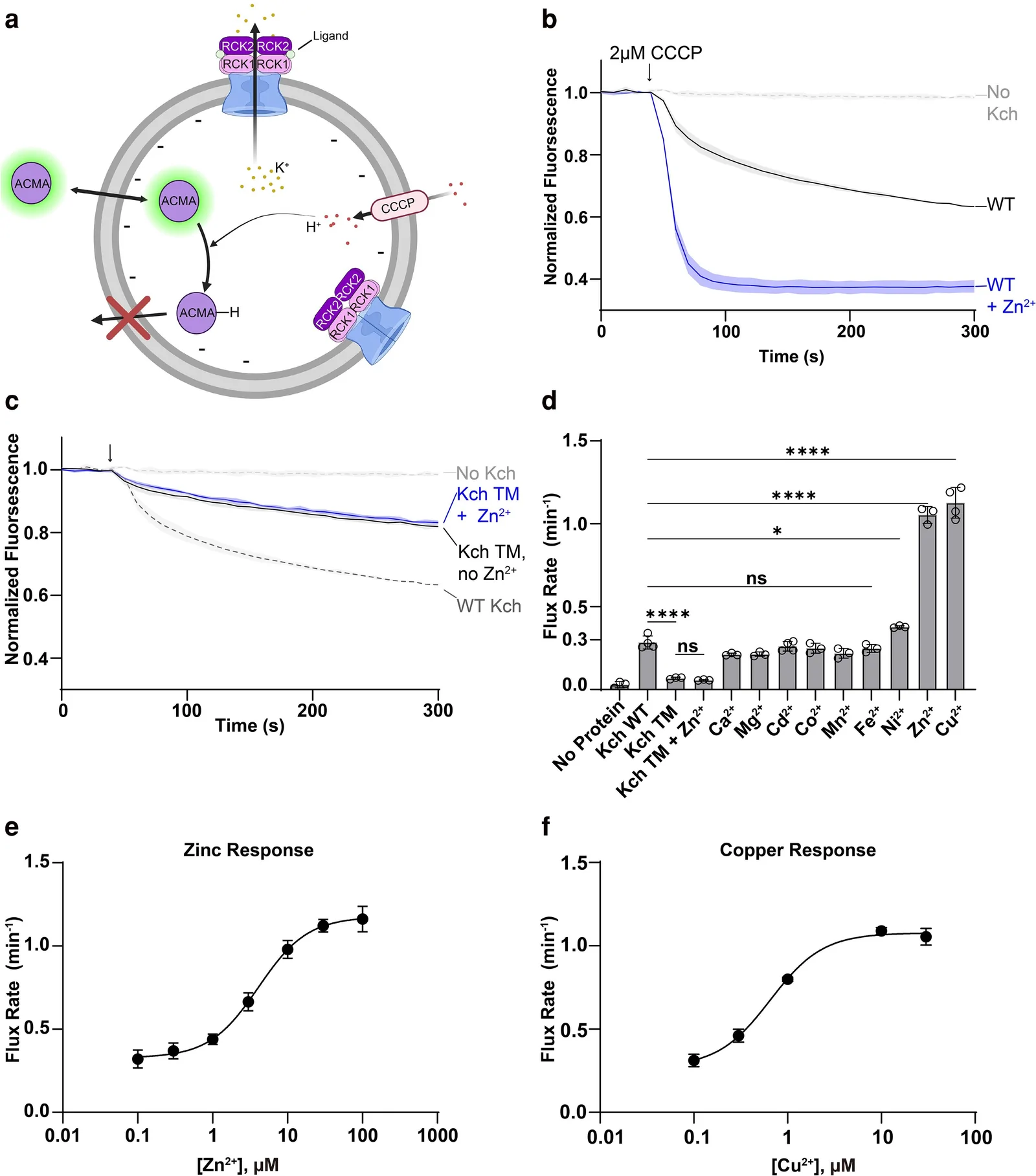
Activation of Kch. a) A schematic drawing of the liposome flux assay. A proteoliposome (gray circles) is shown with Kch in two orientations. Outflow of K^+^ through Kch drives influx of H^+^ through CCCP to quench the fluorescence of amino-chloro-methoxyacridine (ACMA) dye. b) Normalized ACMA fluorescence recorded over time in liposomes with no Kch, and with Kch in the absence or presence of 100 µM Zn^2+^. c) ACMA fluorescence from proteoliposomes containing Kch-TM in the absence (black) or presence of 100 µM Zn^2+^ (blue). d) Flux rate of Kch and Kch-TM in the presence or absence of divalent metal ions. Metal ions were tested at 100 µM except for Cu^2+^ (30 µM). Statistical significance is assessed by one-way ANOVA: ns, not significant; * *P* < 0.05; ****P < 0.0001. e) Flux rates (circles) are plotted at different concentrations of Zn^2+^ and fit to a nonlinear Hill equation (smooth curve) with EC_50_ = 4.1 µM and Hill Slope = 1.3, or f) Cu^2+^ with EC_50_ = 0.65 µM and Hill Slope = 1.6. Throughout this article, time-dependent fluorescence traces are shown as solid lines with shades, in which the solid line represents the mean and the width of the shade represents the SD from three or more independent measurements. In bar graphs, the height is the mean and the error bar the SEM from three or more independent measurements, with individual data points shown as a scatter plot.

Purified Kch was reconstituted into liposomes and its activity was examined using a fluorescence-based flux assay (Figs. 1a and S2, Methods). In the absence of any ligand, Kch has a basal level activity indicated by a modest quench of the fluorescence, and the quench is significant compared to control liposomes that do not have any Kch (Fig. 1b). The basal activity is blocked by nonspecific K^+^ channel pore blocker tetrabutylammonium (29–32) (TBA, Fig. S3a).

To unveil the ligand that activates Kch, we tested known activators of K^+^ channels that are gated by their RCK domains (3, 13, 33, 34). Common nucleotides such as ATP, ADP, NAD^+^, and NADH, and alkali cations such as Ca^2+^ and Mg^2+^, do not affect Kch activity significantly (Figs. 1d and S3f). However, certain transition metal ions such as Zn^2+^ and Cu^2+^ and to a lesser extent Ni^2+^, elevate Kch activity, while other transition metals such as Fe^2+^, Co^2+^, Mn^2+^, and Cd^2+^ have no significant effect (Figs. 1d and S3b, c). Flux rate, as defined by the initial rate of fluorescence change, increased by 4-fold with the addition of either Zn^2+^ or Cu^2+^, and by ∼1.5-fold with Ni^2+^ (Fig. 1d). Further experiments showed that Zn^2+^ or Cu^2+^ activates Kch in a dose-dependent manner with an EC_50_ of ∼4.1 µM and ∼0.65 µM, respectively (Figs. 1e, f and S3d, e, g, h).

To determine whether metal ion activation is mediated by the RCK domain, we expressed and purified Kch-TM, which lacks the RCK domain (Fig. S2). When reconstituted into liposomes, Kch-TM exhibits a reduced yet significant basal activity, however, the activity is no longer enhanced in the presence of 100 µM Zn^2+^ (Fig. 1c, d), consistent with the conclusion that activation of Kch by Zn^2+^ or Cu^2+^ is mediated by the RCK domain. Interestingly, Kch-TM has a lower basal activity compared to that of Kch WT, suggesting that the apo RCK gating ring is partially responsible for the basal activity (Fig. 1c).

### RCK gating ring in three conformations

Expression of the *kch* gene in *E. coli* produces two proteins, the full-length Kch (residues 1–417) and the RCK domain (residues 240–417), and the two assemble into a tetrameric K^+^ channel with an octameric RCK gating ring (9, 11) (Fig. S2). However, the overexpressed RCK domain can also assemble into a standalone ring (Fig. S2, Methods), which interferes with the purification and structure determination processes. We have attempted two ways to eliminate the presence of excessive RCK domains.

First, we made Kch-2xRCK, which has a M240L mutation to eliminate the alternative start codon, and a second RCK domain spliced to the C-terminus of the first (Fig. S1c and Methods). As expected, this construct produces a tetrameric Kch-2xRCK channel, which is stable and has comparable response to Zn^2+^ as that of the wild-type Kch (Figs. S2 and S3i, j). We collected a cryo-electron microscopy (cryo-EM) dataset of Kch-2xRCK in the absence of Zn^2+^ or Cu^2+^, and after particle extraction, 2D classification and 3D reconstructions, two density maps emerged, in which the RCK gating ring is well-resolved but the transmembrane domain is only partially resolved (Fig. S4). Additional data processing procedures improve the resolution of the TM domain, with transmembrane helices 3–6 becoming recognizable, but the TM region remains insufficiently defined for building a structural model (Fig. S5). We will thus focus our structural analysis on the RCK gating ring.

The two density maps show distinct conformations of the octameric RCK gating ring, one dilated and square and another rectangular shaped. Further refinements, which are detailed in Methods and shown in Fig. S4b, lead to density maps of the RCK ring at a nominal resolution of 2.9 Å (Figs. S4, S6, and S7). We define the most dilated conformation as the open conformation, and the rectangular elongated conformation as the intermediate.

**Figure 2:**
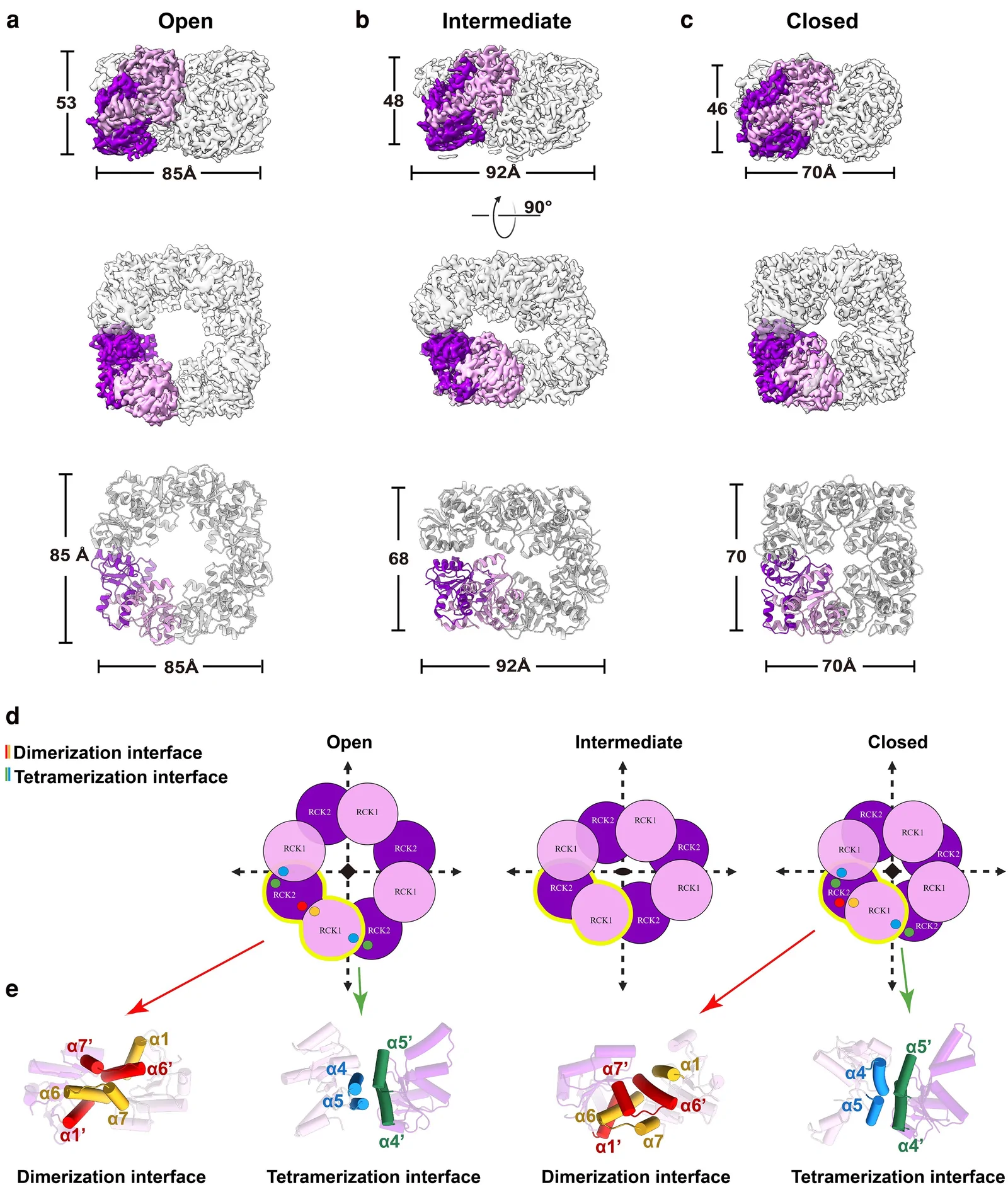
Three conformations of the RCK ring. a–c) Cryo-EM maps of three RCK conformations viewed from the side (top panels) and along the membrane norm (middle panel), and the structure models of the rings (bottom panels). A single RCK dimer is colored. d) Schematic assembly of RCK gating rings in open (left), intermediate (middle), and closed (right) conformations. A single RCK dimer is traced with a yellow line. Colored dots represent the dimerization (red and orange) and tetramerization (green and blue) interfaces between RCK domains in RCK gating ring. e) The interface between the two RCK domains within a dimer and between two adjacent RCK dimers in cylinder representation.

The open RCK gating ring has a C4 symmetry and dimensions of ∼ 85 × 85 × 53 Å (Fig. 2a). Four RCK dimers assemble around the 4-fold symmetry axis. In each RCK dimer, the two RCK domains are almost identical with a backbone root-mean-square deviation (RMSD) of 0.2 Å. Each RCK monomer has seven alpha helices (α1–7) and six beta strands (β1–6), forming an α4/β6 Rossmann-like fold that has three extra helices, one in between β3 and β4, and two (α6 and α7) forming the HLH at the C-terminus (Fig. S8). The dimer interface is mainly composed of the HLH motif from each RCK monomer and with minor contributions from αl. In the open conformation, the neighboring RCK dimers make contact through four identical tetramer interfaces, which are formed by α4 and α5 (Fig. 2d and e).

The intermediate RCK gating ring has 2-fold symmetry with dimensions of ∼ 92 × 68 × 48 Å (Fig. 2b). Two of the diagonally opposing RCK dimers are identical to the RCK dimers in the open gating ring, which we term the open RCK dimer, while the other two RCK dimers have a different conformation, and we define them as the closed RCK dimers for reasons that will become clear below. Consequently, the intermediate RCK gating ring has two different tetramer interfaces that result in an elongated rectangular shape (Figs. 2d, e and S9).

Second, excessive RCK domains produced in expression of the wild-type Kch can be reduced significantly by isolating the cell membrane before detergent solubilization and purification of the channel (Fig. S2 and Methods). To visualize the effects of Zn^2+^ on the RCK gating ring, we collected a dataset of the wild-type Kch in its presence (0.5 mM). Presumably due to the presence of Zn^2+^, we did not observe channels with gating rings in the intermediate conformation during 2D classification, and the open RCK gating ring was the sole dominant species in this dataset, which lead to a structure determined at a resolution of 3.2 Å (Figs. S10 and S11).

In addition to single Kch channel particles, a peculiar “tail-to-tail” Kch channel dimer was present in the same data set (Fig. S10a). By intentionally selecting the tail-to-tail channel dimer particles during the data processing, we obtained a structure of the RCK gating ring in a third conformation at ∼2.9 Å, which is more compact and we define it as the closed RCK gating ring (Figs. S10 and S12). The closed RCK gating ring maintains the C4 symmetry and has dimensions of 70 × 70 × 46 Å (Fig. 2c). All four RCK dimers in this conformation are almost identical to the closed RCK dimers found in the intermediate RCK gating ring. The interface between the two channels is formed by residues at the N terminus of the free RCK domain (Fig. S13d–f), residues H241 to H247. While the same residues in the first RCK domain are part of the linker to the TM domain, they are untethered in the second RCK domain and mediate the interactions of the two gating rings.

### Assembly of the RCK dimers

Structural changes between the open and closed RCK gating ring can be described at three levels, the individual RCK monomer, the RCK dimer interface, and the tetramerization interface between the RCK dimers.

**Figure 3:**
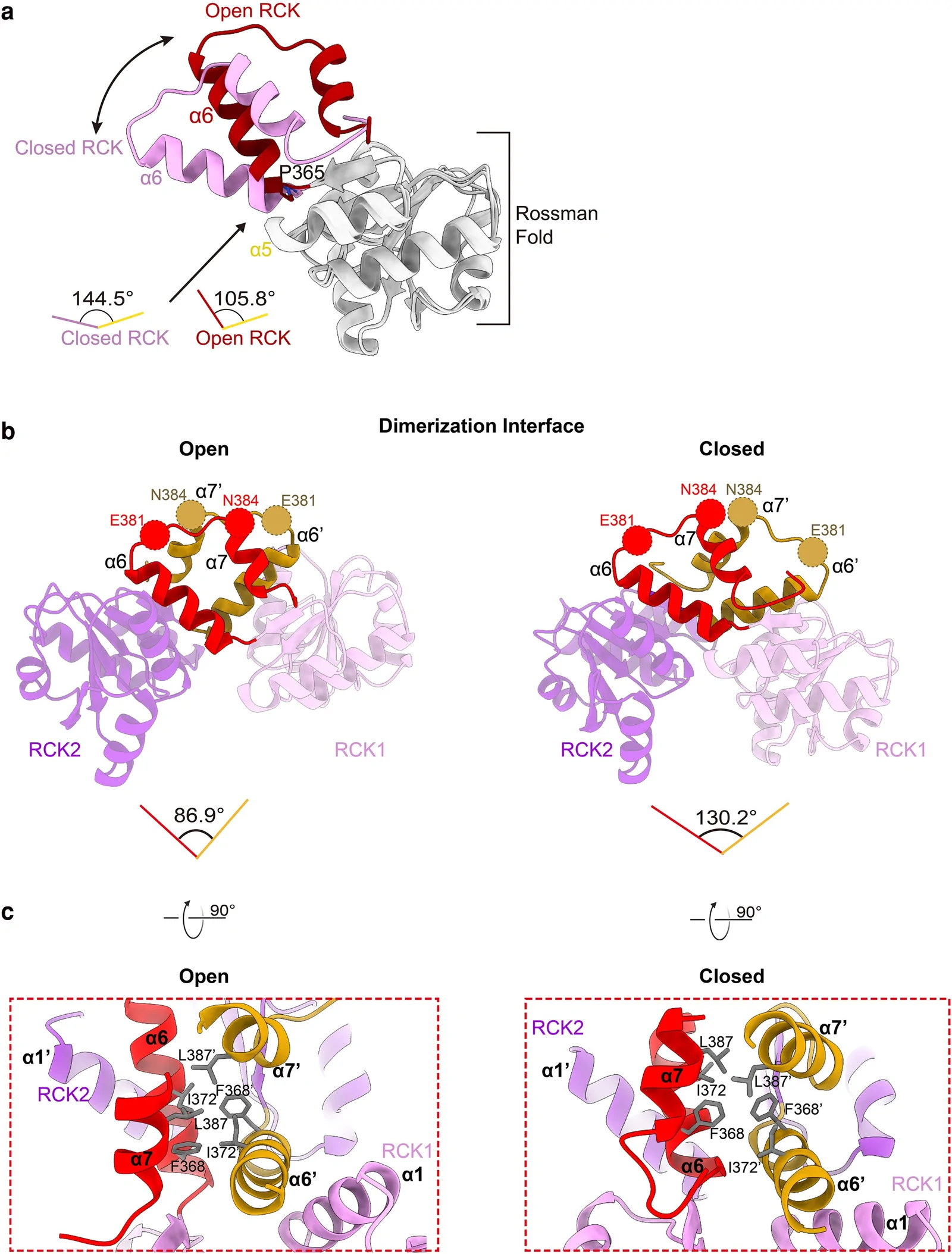
The open and closed RCK dimers. a) Cartoon depiction of an RCK monomer highlighting the movement in the C-terminus HLH motif between the open (deep red) and closed (light pink) conformations. Angles represent the change in orientation between α6 and α5, with P365 as the vertex. b) Cartoon depiction of the RCK dimerization interface where α6 & α7 from RCK1 (red) and α6' & α7' from RCK2 (orange) are compared in the open (left) and closed (right) RCK conformations. Angles represent the scissoring motion of the dimer interface, illustrated as the angle change between α6 (red) & α6' (orange). c) Zoomed-in view of the hydrophobic dimerization interface in the open (left) and closed (right) RCK dimers.

The RCK monomers from the open and closed RCK dimers have different conformations, in which the Rossmann fold and the HLH motif remain rigid and can individually be well-aligned, with an RMSD of ∼0.5 Å. However, there is a pivot of the HLH motif, centered around residue P365, relative to the Rossmann fold (Fig. 3a). In the open RCK monomer, the angle between the HLH motif and the Rossmann fold, defined as the angle between helices α5 and α6, is ∼105°, while that of the closed RCK monomer, ∼145° (Fig. 3a).

The RCK dimer interface is mainly composed of the two HLH motifs forming “hand-shake” like interactions. In the open RCK dimer, the angle between the two HLH motifs is ∼87°, while in the closed conformation, ∼130° (Figs. 3b and S14a). While the open RCK dimer aligns well to the previously determined X-ray structure, the closed RCK dimer aligns well within the Rossmann fold but differs substantially in the dimer interface (Fig. S15). The differences between open and closed RCK dimers are most dramatic at the loop of the HLH motif, where residues N384 from one monomer and E381 from another one are within hydrogen bond distance in the open conformation, but are 15 Å away in the closed conformation (Figs. 3b and S14a). Nevertheless, the scissoring movement of the HLH motifs does not significantly alter the patch of hydrophobic interactions that form the bulk of the dimer interface (Fig. 3c).

### Assembly of the RCK gating ring

**Figure 4:**
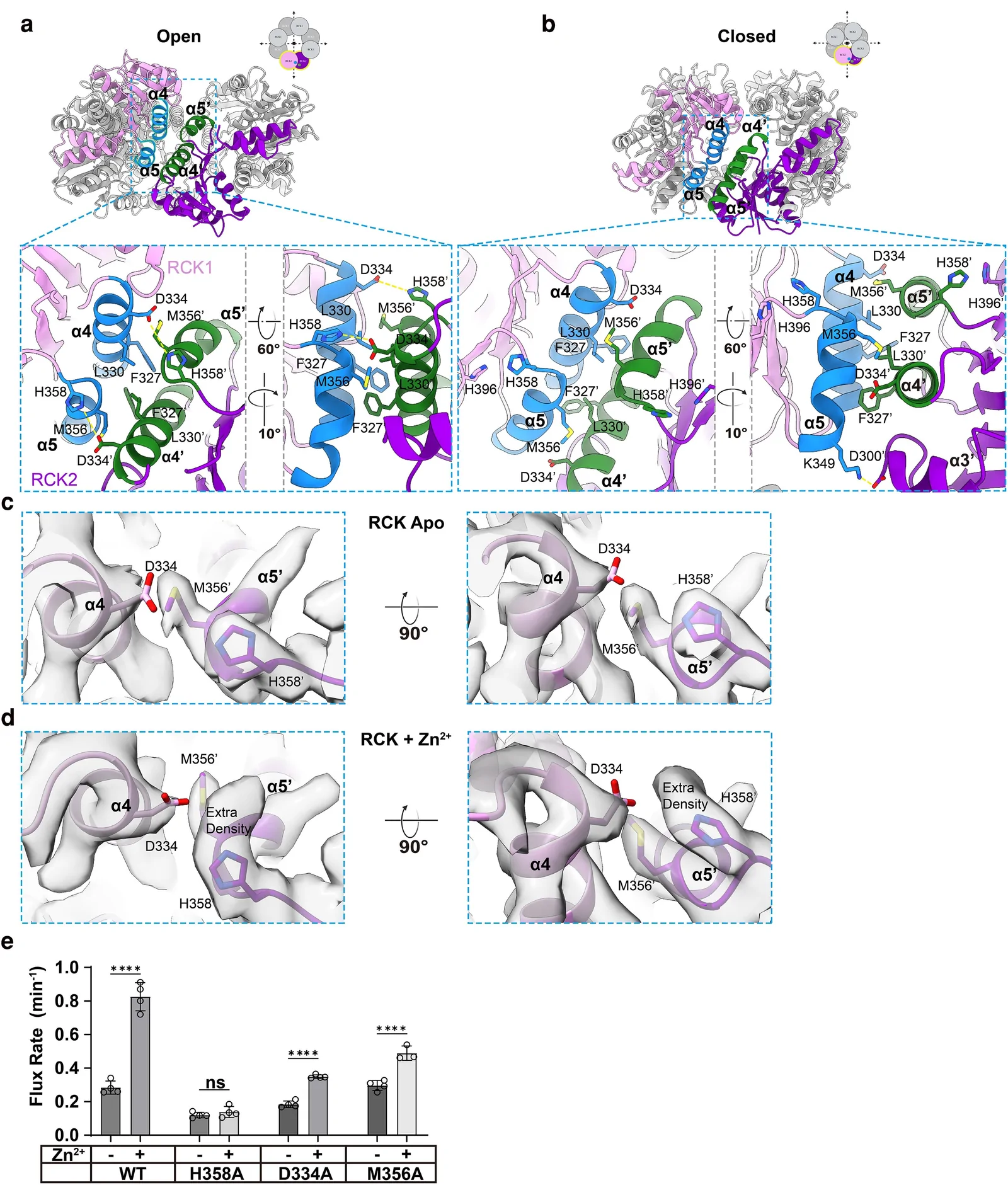
The RCK tetramerization interface. The tetramerization interface of an RCK octameric gating ring in the open (a) and closed (b) conformations. The interacting helices are shown in blue and green cartoon, and the interface residues are shown in sticks in two orientations (cutout views). c) Top and side views of the tetramerization interface between two RCK dimers in the apo sample or d) in the presence of Zn^2+^, with annotated Zn^2+^ binding residues and an extra density upon addition of Zn^2+^ to the sample. e) Flux rates of wild-type and mutant Kch. Comparisons are performed using one-way ANOVA: ****P < 0.0001.

Four RCK dimers assemble into a gating ring through interactions between the first RCK domain of one dimer and the second RCK domain from the neighboring dimer (Fig. 2d, e). The tetramerization interface involves α4 and α5 from neighboring RCK domains (Figs. 4a, b and S14b, c), in which pairs of F327 and L330 form a hydrophobic core. Several hydrophilic interactions are found in the periphery of the hydrophobic core (Fig. 4a, b). The assembly of the RCK gating rings follows the same principles seen in other RCK rings, although the residues at the interface differ in different channels.

In the open gating ring, the angle between the interface helices, α4 and α4', is 65°, while that in the closed gating ring is 102° (Fig. S14d). The change brings the ring diameter from ∼120 Å in the open conformation to ∼99 Å in the closed conformation (Fig. 2b, c). Interestingly, hydrophobic interactions at the tetramer interface remain relatively intact in the two conformations, while the hydrophilic residues at the ends of the two helices experience a larger separation. Particularly, H358 from one RCK dimer and D334 from the neighboring RCK dimer are ∼4.5 Å in the open conformation but ∼ 14 Å apart in the closed conformation (Fig. S14d).

The open RCK gating ring in the presence of Zn^2+^ has a density near the H358–D334–M356 triad at the tetramer interface, and this density has a σ level comparable to surrounding residues and it is not present in the apo open RCK gating ring (Fig. 4c, d). Although H358 seems to coordinate the putative Zn^2+^, we hesitate to identify this as a Zn^2+^ due to the modest local resolution and the lack of carbonyl density from D334. We made mutations to candidate Zn^2+^ binding sites throughout the RCK domain, and H358A stood out as the only one that eliminates Zn^2+^ activation (Fig. 4e), while others have either partial or no effect on Zn^2+^ activation (Fig. S16). Importantly, the putative binding site triad is only formed in the open conformation of the tetramerization interface. When also considering that presence of Zn^2+^ drives the majority of the RCK gating rings to the open state, we propose that Zn^2+^ promotes the open RCK gating ring by binding to the tetramer interface.

## Discussion

In summary, we expressed and purified Kch and demonstrated the activity of purified Kch in a liposome-based flux assay. We discovered that both Zn^2+^ and Cu^2+^ significantly increase the activity of Kch, and showed that common ligands known to activate channels with RCK domains, such as Ca^2+^ or nucleotides, do not activate Kch. We captured the structure of the RCK gating ring in three conformations, closed, intermediate, and open, which led us to propose a mechanism of Kch gating controlled by the expansion and contraction of the gating ring.

We start with the assumption that the gate of Kch is formed by the C-terminus of the pore-lining helix (TM6) in an iris like constriction (16, 35). The linker that connects TM6 and the RCK domain would mechanically transmit the change in the diameter of the gating ring to changes at the gate of the channel. Comparing the RCK gating ring between the open and closed conformations (Fig. 2a, c), the diagonal diameter of the Kch gating ring increases by ∼20 Å in the open state, sufficient to dilate the gate to allow permeation of hydrated K^+^, which has a diameter of ∼7 Å (36). However, the details of the mechanism require further structural studies to fully resolve the linker and the TM domains.

**Figure 5:**
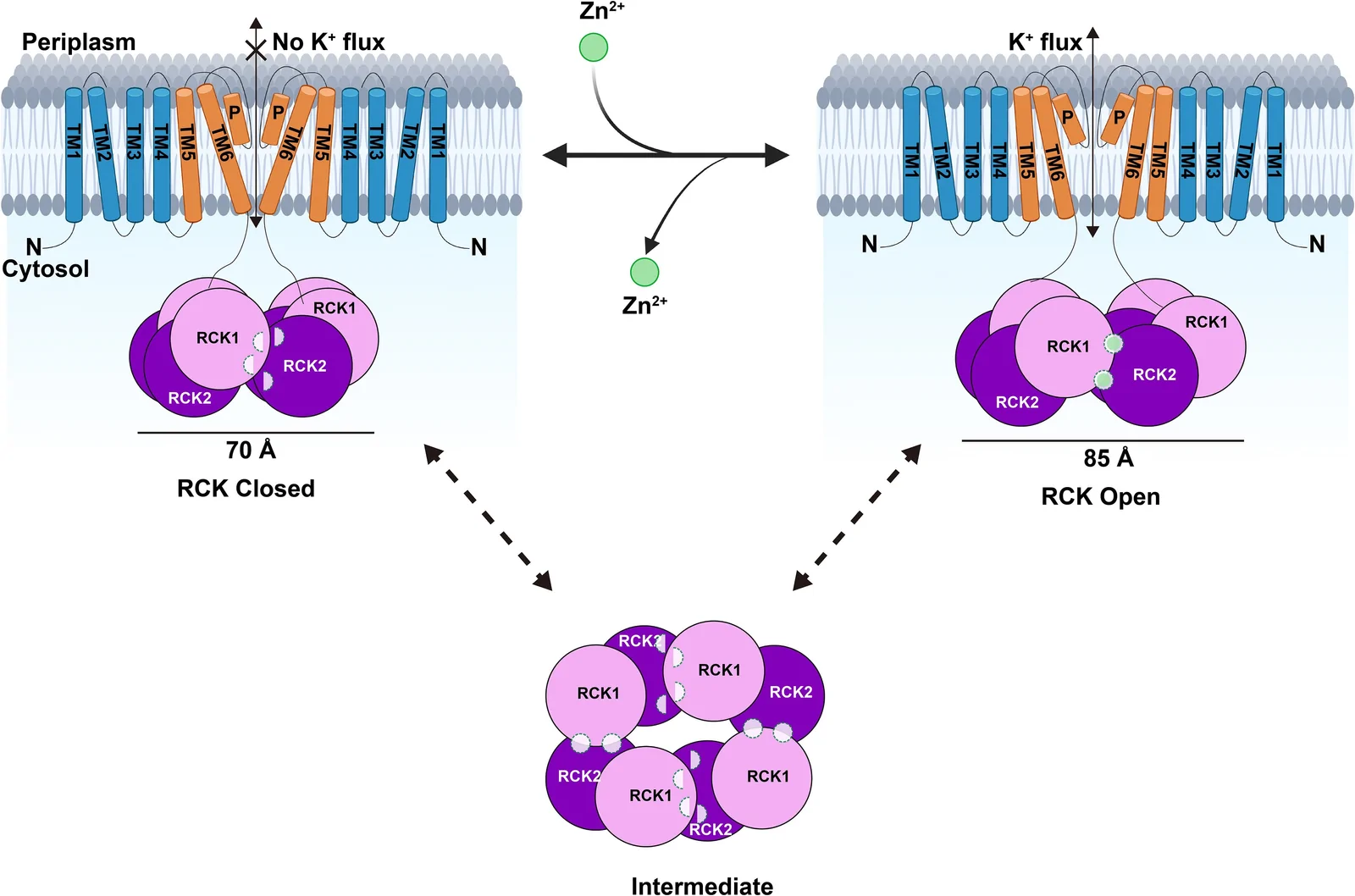
Proposed mechanism for zinc-dependent activation of Kch. Kch channel shown with two diagonally opposing membrane-embedded subunits and a complete gating ring. Left, channel in the closed state with a compact 4-fold symmetric RCK gating ring; Right, channel in the open state with a 4-fold symmetric dilated open RCK gating ring upon Zn^2+^ binding. An intermediate 2-fold symmetric RCK ring is shown at the bottom. The intermediate ring is likely a required step in activation.

Since we observed fully formed putative Zn^2+^ binding sites in the open conformation and the complete disruption of these sites in the closed conformation, we speculate that the open and closed RCK rings correspond to open and closed Kch channel (Fig. 5). We further conclude that Zn^2+^ stabilizes the open RCK ring conformation. However, we are less certain about the correlation between the intermediate conformation of the RCK gating ring to channel activity. In the intermediate conformation, the gating ring is composed of two open RCK dimers and two closed RCK dimers, and we do not have sufficient data to determine if the channel is in a conducting state.

Structural changes in the gating ring of Kch are consistent with these of previously reported eukaryotic and prokaryotic K^+^ channels with gating rings (16, 26, 27, 37). The conformational changes share common features at both the dimerization and tetramerization interfaces. The gating ring is composed of a tetramer of RCK dimers, and whether the RCK dimer is composed of two identical domains, as in the case of Kch, or different RCK domains, the changes at the dimer interface seem to involve a scissoring motion while the changes at the tetramer interface involve a slippage (Figs. 3, 4, and S14). The intermediate conformation was not previously observed in related K^+^ channels, and it suggests a potential mechanism that requires a sequential change of the ring conformation during the gating process.

When the wild-type Kch is overexpressed in *E. coli*, there is a substantial amount of standalone RCK domains, which form soluble RCK gating rings and can polymerize to form fibers. We determined the structure of the RCK rings in the fibers and found that these rings are composed of five RCK dimers (Fig. S17). The construction of the decameric RCK ring is similar to that of the open octameric gating ring in that each RCK dimer assumes the open conformation, however, the assembly interface between the neighboring α4 and α5 helices have a more acute angle. As for the interface mediating the stacking of the decameric rings, it is formed by the N terminus of the RCK domain, and similar to the “tail-to-tail” Kch dimer structure (Fig. S17). In both cases, H241 seems to be important for the interface, and interestingly, the H241A mutant noticeably reduces the fraction of free RCK rings in the purified protein and eliminates RCK fibers on grids (Fig. S13).

Kch has been implicated in metabolic control of porin permeability and antibiotic susceptibility in *E. coli*. Loss of Kch increased resistance porin-dependent antibiotics, indicating that Kch activity can influence antibiotic entry into the cell (6). These findings highlight the physiological relevance of Kch and provide important context for the present study. In this framework, our identification of Zn^2+^ and Cu^2+^ as direct agonists of Kch suggests a plausible biochemical route by which Kch activity could be enhanced to regulate porin permeability, and thereby may help guide future efforts to understand how modulation of Kch activity influences antibiotic susceptibility in bacteria at a molecular level.

Is Zn^2+^, Cu^2+^, or Ni^2+^ a native ligand to Kch? While the overall amount of Zn^2+^ in an *E. coli* is in the millimolar range, nearly all of it is sequestered in protein bound form leaving virtually no free Zn^2+^ in the cytoplasm (38). Most of the Cu^2+^ is also protein bound (39, 40). Similarly, although Ni^2+^ is overall more abundant, its concentration is also tightly buffered and free Ni^2+^ concentration is low (41). Free Zn^2+^ concentration in *E. coli* may rise transiently as a part of the bacterial stress response (42), but the concentration is not known to surpass the nanomolar range, which is not sufficient for Kch activation. We imagine that a more likely scenario would involve a metal ion chaperone delivering the ions directly to the vicinity of the gating ring. Interestingly, even though free Zn^2+^ is extremely scarce in eukaryotic cells as well, it was also shown to enhance activation of BK channels through binding to the RCK domains (43). Regardless, our demonstration of Kch function, identification of transition metal ions as a new class of RCK-activating ligands, and analysis of the structural plasticity of the RCK gating ring brings us a step closer to understand the role of Kch in *E. coli.*

## Methods

### Protein expression and purification

The full-length Kch gene (Uniprot ID: P31069) from *E. coli* was cloned into a modified pET vector with a tobacco etch virus (TEV) protease cleavage site, followed by a 10-polyhistidine tag on the C-terminus of the open reading frame. For Kch-2xRCK, a second RCK (AA 240–417 of the full-length sequence) was spliced into the C-terminus of the first via a GSGS linker of four residues. The last 30 residues of the RCK C-terminus are not structurally resolved and thus likely flexible, which should not constrain the placement of the second RCK.

Each vector was transformed into *E. coli* BL21(DE3) strain and cultured in LB medium supplemented with 50 µg/mL kanamycin at 37 °C. When cell growth reached an optical density of ∼0.7 (600 nm), protein expression was induced by 1 mM isopropyl β-d-1-thiogalactopyranoside for 4 h at 37 °C. The cells were harvested, and the pellets were flash frozen and stored at −80 °C.

Cells were lysed by gentle sonication in 150 mM KCl, 20 mM HEPES, and 10% glycerol, 1 mM phenylmethylsulfonyl fluoride, 2 mM β-mercaptoethanol, 5 mM MgCl, and DNAseI. To purify Kch, the lysate was supplemented by 1% (w/v) Lauryl Maltose Neopentyl Glycol (LMNG) and rocked in room temperature for 2 h to solubilize membrane proteins. The cell lysate was then cleared by centrifugation at 55,000 × *g* for 45 min, and the supernatant was loaded onto TALON Metal Affinity Resin packed in a gravity column pre-equilibrated with column buffer A (150 mM KCl, 20 mM HEPES, and 10% glycerol, 0.1%, and 2 mM BME), and subsequently washed by 10 column volume (CV) of column buffer A and 10 CV of column buffer A supplemented by 20 mM imidazole. The protein was eluted from the resin by TEV cleavage (1 mg TEV, 1 h room temperature) and further purified by size-exclusion chromatography on an SRT-10C column in 150 mM KCl, 20 mM HEPES, 0.01% LMNG, or 0.02% GDN, or no detergent. The fractions containing the fully assembled tetrameric channel were analyzed by SDS-PAGE, pooled together and concentrated for either liposome reconstitution or structure determination.

For the membrane prepared wild-type Kch with the presence of Zn^2+^ sample, cell lysate from 9L of cells was lysed and centrifuged at 100,000 g for 2 h before solubilization to get rid of excess soluble RCK. The membrane pellet was then resuspended and followed the same protocol as stated above.

For the soluble RCK sample, detergent solubilization step was removed from the protocol and subsequent detergent-containing steps were modified to omit detergent utilization.

Kch mutants were expressed and purified using the same protocol as above.

### Mutagenesis

Kch mutants were generated using the QuickChange method (Qiagen) with slight modifications. The entire cDNA was sequenced to verify the mutations. Primers are available in Table S2.

### Cryo-EM grids preparation and data processing

Cryo grids were prepared on the Thermo Fisher Vitrobot Mark IV. Quantifoil R1.2/1.3 Cu grids were glow-discharged using the Pelco Easyglow. About 3.5 μL of 8 mg/mL Kch was applied to glow-discharged grids. After blotting with filter paper (Ted Pella) for 3.5–4.5 s, the grids were plunged into liquid ethane cooled with liquid nitrogen. For the Kch WT dataset, 0.5 mM Zn^2+^ was added to the sample before blotting.

In data collection, movies were collected on a 300 kV Titan Krios with a Quantum energy filter (Gatan), and at a magnification of ×81,000 and with defocus values of −2.0 to −0.8 μm. A K3 Summit direct electron detector (Gatan) was paired with the microscope. Movies were collected in super-resolution mode with an exposing time of 0.175 s per frame for a total of 50 frames and a total dose of about 50 e^-^ per Å^2^. The movies were motion corrected with Relion 4 and binned (2 × 2) so that the pixel size was 0.832 Å (Texas A&M LBSD) or 1.1 Å (SLAC) and imported into CryoSPARC for further processing (44, 45).

For the Kch-2xRCK dataset, 18,534 micrographs were collected, of which around 11% were excluded based on a CTF resolution limit of 4.5 Å, and a defocus limit of 30,000 Å (46). Initially, blob picking followed by two rounds of 2D classification were used to generate suitable templates for template picking, which then generated 18,170,630 particles. After three rounds of 2D classification, 1,814,511 high quality particles were used for ab initio reconstruction to generate ten 3D classes that were a mixture of junk classes, low quality classes with recognizable full-length channel, and two high quality classes centered around an open and intermediate conformation of the RCK gating ring. These two classes were further processed independently by heterogeneous refinement with a final particle count of 197,637 for the open RCK conformation and 292,104 for the intermediate RCK conformation. The open RCK conformation was nonhomogenously refined with C4 symmetry yielding a map at 2.9 Å resolution (47, 48). The intermediate conformation was nonhomogenously refined with C1 symmetry first and then underwent local refinement with a mask around the RCK domain to minimize noise from the poorly resolved TM domain, and this refinement produced a final map at 2.9 Å resolution.

To obtain the TM density seen in Fig. S5, 1,814,511 particles from the 2D classification step were used in 3D classification of an open, intermediate, or closed ring volumes, without any symmetry enforced. The class belonging to the intermediate conformation of the ring, boxed on the right, showed the best density around the transmembrane domain. The 510,508 particles in this class were used for high-resolution ab initio reconstruction of three classes with C1 symmetry (initial resolution: 12 Å, maximum resolution: 3 Å, Fourier radius step: 0.01, initial minibatch size: 100, final minibatch size: 300, class similarity 0). The class with the best TM density, boxed on the left, was further selected, containing 252,641 particles. After heterogeneous refinement, and without any symmetry enforcement, the TM density was already vaguely recognizable, but enforcing C4 symmetry during nonuniform refinement further enhanced the quality of the map inside the detergent micelle.

For the wild-type Kch-Zn^2+^ dataset, 5,394 micrographs were motion corrected as described above. Around 18% of motion corrected micrographs were excluded based on CTF resolution fit and defocus limits stated above. Blob picking in a small subset of 1,500 micrographs was initially used alongside three rounds of 2D classification to generate high quality templates for template-based picking centered around the Kch channel dimer. Template picking generated 1,560,531 particles, which after five rounds of 2D classification resulted in 82,069 high quality particles. These particles were used for ab initio reconstruction to generate three 3D classes. The volumes were then refined using heterogeneous refinement and the best class, containing 55,071 particles, was refined with C4 symmetry nonhomogenous refinement producing a map of an RCK octamer dimer or “tail-to-tail” channel dimer at 3.06 Å resolution. This map was used to build a model of the two RCK rings, as seen in Fig. S13. The map was further refined locally with a mask around the top RCK octamer, which was slightly better resolved than the bottom layer. The final refined map had a resolution of 2.88 Å.

For the open RCK structure in presence of Zn^2+^, the tail-to-tail dimers were excluded during initial 2D classification, leaving around 560,866 single channel particles. These particles were used to generate four ab initio 3D classes, and heterogeneous refinement was performed with the forementioned classes plus two junk volumes to remove low quality particles. This step was repeated with the particles belonging to the heterogeneous refinement class showing the best RCK gating ring density. The final 185,079 particles were used for nonuniform refinement with C4 symmetry, which yielded a map of an open RCK octamer at 3.2 Å resolution.

For the RCK fiber structure, the dataset contained 9,885 micrographs, which were motion-corrected as described above. In CryoSPARC, around 1% of the micrographs were designated outliers and excluded based extreme CTF fit resolution or defocus values. Blob picking was done in the entire dataset, which yielded 4,531,761 particles, most of which were clearly recognizable fibers. Extensive 2D classification yielded a final count of 261,166 fiber particles, which were then used as templates for the filament tracer job. Filament tracer yielded around 1,889,050 particles, which further underwent 2D classification until a final count of 197,803 best-looking particles was selected. Particles were re-extracted at a 400px box size with 2-fold binning. Helical refinement with C1 symmetry resulted in a clearly recognizable 5-fold symmetric fiber particle at a resolution of ∼4.4 Å. The helical refinement job was repeated by enforcing C5 symmetry, which improved the resolution to around ∼3.7 Å. Symmetry search was done to find the optimal twist and rise values present in the fiber. Particles were re-extracted without binning, and helical refinement with C5 symmetry was performed with the adjusted rise and twist values, yielding a map of five stacked decameric RCK gating rings at a resolution of 3.6 Å. To improve the map quality around the ring stacking interfaces, a local refinement mask was created around the two best resolved rings, which improved the map to around ∼3.3 Å resolution.

### Model building and refinement

The RCK gating ring in the open conformation from the Kch-2xRCK dataset was built by using the previously determined RCK dimer crystal (PDB:1ID1) (8). For the RCK gating ring in the intermediate conformation, a combination of the RCK dimer crystal and an AlphaFold multimer prediction model were used as a starting point to build the oligomer (49, 50). The models were manually adjusted in Coot, then PHENIX was used to subject the RCK dimer to NCS symmetry operations according to map symmetry and real-space refinement (51, 52). Map and models depicted via PyMol (The PyMol Molecular Visualization System, Version 1.8, Schrodinger, LLC) or ChimeraX (53).

For the RCK gating ring in the closed conformation and the “tail-to-tail” dimers structures determined from the wild-type Kch-Zn^2+^ dataset, an initial model of the RCK domain from an AlphaFold multimer prediction was used. The model was refined as described above.

For the RCK gating ring in the open conformation from the wild-type Kch-Zn^2+^ dataset, the RCK dimer crystal (PDB:1ID1) was used as initial model, and the rest of the process was the same as described above.

For the decameric RCK fibers, the starting point for model building was the open RCK dimer. A single dimer from the open RCK gating ring structure was manually fit into the decameric fiber density, then refined manually using Coot. Phenix was used to compute Map Symmetry and apply NCS operators to the resulting dimer, converting the single model to a 5-fold symmetric ring. Real-space refinement was used to further optimize the decameric model. Upon refinement, the ring model was duplicated and inverted to generate a mirror image of the bottom ring in the two-ring stack, and fit into the top ring density to create the model of two RCK rings stacked in the fiber.

### Liposome reconstitution

Phosphatidyl ethanolamine (Avanti) and phosphatidyl glycerol (Avanti) were mixed in a 3:1 weight ratio, dried under argon and further vacuumed for 1 h to remove residual chloroform. Lipids were resuspended in 150 mM KCl, 20 mM HEPES (pH 7.5) to 10 mg/mL final concentration and sonicated to clarity before addition of TritonX-100. Mixture was shaken at 90 rpm at room temperature for 15 min before the addition of protein at a final ratio of 1:100 protein:lipids (w:w). For the empty control, the same volume of blank buffer was added. Mixture was incubated under gentle shaking for 30 min at room temperature. For two cycles, around 60 mg Semiwet biobeads (Sm2 Bio-Rad) were added per 1 mL of mixture and incubated for 1 h under gentle shaking at room temperature. In the final step, 120 mg semiwet biobeads were added per 1 mL of mixture and incubated at 4 °C under gentle shaking overnight. The liposomes were harvested and flash frozen, stored in −80 °C until use.

### Flux assay

The assay set up was similar to SK Lee et al. (54). Liposomes were thawed, diluted 2-fold with 150 mM KCl, 20 mM HEPES (pH 7.5), and frozen-thawed three times. Liposomes were extruded to homogeneity with a 400 nm filter (NanoSizer Extruder, T&T Scientific Corporation). Liposomes were then diluted 20-fold into a buffer containing 150 mM NaCl, 20 mM HEPES pH 7.5), and 2 µM 9-amino-6-chloro-2-methoxyacridine dye (ACMA). After a 5-min incubation, fluorescence was monitored in a quartz cuvette at room temperature using the FluoroMax-4 spectrofluorometer (HORIBA) with 410 nm excitation and 477 nm emission at 10 s intervals. Proton influx was initiated by the addition of 2 µM carbonyl cyanide m-chlorophenyl hydrazone (CCCP), and ACMA fluorescence quenching was monitored over time. The initial flux rate is defined as the slope of a linear fit to the first 40 s of data points immediately after the addition of CCCP.

## Supplementary Material

pgag284_Supplementary_Data

## Data Availability

The atomic coordinates of the Kch RCK gating ring in the open, intermediate, and closed conformations have been deposited in the PDB (http://www.rcsb.org) under the accession codes 9YE0, 9YFV, and 9YGL, respectively. The atomic coordinates of the Kch RCK gating ring in the open conformation in the presence of Zn^2+^ have been deposited under the accession code 9YKQ. Their corresponding electron microscopy maps have been deposited in the Electron Microscopy Data Bank (https://www.ebi.ac.uk/pdbe/emdb/) under the accession codes EMD-72842, EMD-72908, and EMD-72932 for the open, intermediate, and closed RCK rings, respectively. The electron microscopy map for RCK gating ring in the open conformation in the presence of Zn^2+^ has been deposited under accession code EMD-73056. Source data are provided with this article.
