# Supplementary material for: Mechanism of Kch activation by Zn2+
Source: PNAS Nexus. 2026 Aug 31;5(9):pgag284. doi: 10.1093/pnasnexus/pgag284 (PMC13563188; doi:10.1093/pnasnexus/pgag284)
Supplement: Supplementary file 1 [file pgag284_supplementary_data.docx]

**Supplementary Figures:**

**S1 Fig. *Kch* gene arrangement and tetrameric channel assembly. a.** Cartoon depiction of the *kch* gene, with a main start codon that translates a full-length TM-RCK protein, and an alternate start codon that results in standalone expression of the soluble RCK domain. **b.** Assembly of a functional tetrameric Kch channel composed of 4 copies of the full length TMD-RCK and four copies of the standalone RCK domain. **c.** Cartoon depiction of the Kch2xRCK construct, where the alternate start codon is eliminated via the M240L mutation, and where a second RCK domain is spliced into the C terminus of the first via a linker.

**S2 Fig. Protein Purification.** Size-exclusion chromatography profiles of different Kch constructs alongside the SDS-PAGE analysis of the selected purified products. Red bar on the size exclusion profile represents the fractions analyzed, either shown as individual lanes or pooled together and concentrated.

**S3 Fig. Kch function, zinc & copper dose response and mutant activity. a.** Traces of ACMA fluorescence quench over time in liposomes with no Kch (gray), Kch proteoliposomes in the absence of ligand (Black), or in the presence of 0.1mM Zn^2+^. At 420s, 450s, and 510s different amounts of TBA was added, which blocks the pore on the cytosolic side and reduces K^+^ outflux flux, which in turn results in a diminished proton influx and causes ACMA deprotonation and fluorescence de-quenching. **b.** Fluorescence quench over time in liposomes with no Kch (gray), Kch proteoliposomes in the absence of ligand (black), presence of 0.1 mM Mn^2+^(purple), Fe^2+^(pink), Co^2+^(maroon), Cd^2+^(dark yellow), Ni^2+^(green), Zn^2+^(blue), or 30 μM Cu^2+^(red). **c.** Fluorescence quench over time in liposomes with no Kch (gray), Kch proteoliposomes in the absence of ligand or chelator (black), presence of 2 mM EDTA (light pink), 2 mM EDTA + 0.1 mM Zn^2+^(blue) or only 0.1 mM Zn^2+^ (dark pink). **d**. Fluorescence quench over time in liposomes with no Kch (gray), Kch proteoliposomes in the absence of ligand (black), presence of 0.1 μM Zn^2+^ (purple), 0.3 μM Zn^2+^ (orange), 1 μM Zn^2+^ (blue), 10 μM Zn^2+^ (green), 30 μM Zn^2+^ (brown), or 100 μM Zn^2+^(magenta). **e.** Fluorescence quench over time in liposomes with no Kch (gray), Kch proteoliposomes in the absence of ligand (black), presence of 0.1 μM Cu^2+^ (purple), 0.3 μM Cu^2+^ (orange), 1 μM Cu^2+^ (blue), 10 μM Cu^2+^ (green), or 30 μM Cu^2+^ (brown). **f**. Fluorescence quench over time in liposomes with no Kch (gray), Kch proteoliposomes in the absence of ligand (black), presence of NADH (green), NAD^+^ (blue), ATP (orange), or ADP (purple).**g.** Raw Fluorescence quench data for three dose response concentrations shown in **d**. **h.** Raw fluorescence quench data for three dose response concentrations shown in **e**. **i** Fluorescence quench of Kch 2xRCK proteoliposomes in the absence (black) or presence (red) of 0.1 mM Zn^2+^. **j** Flux rate for experiments shown in **i**. Two-tailed Welch *t*-tests were applied for comparison. ** indicates *p* < 0.005.

**S4 Fig. Cryo-EM data processing of the open & intermediate conformation RCK rings from** **Kch2xRCK**. **a**. A representative micrograph of Kch2xRCK (Left), its Fourier transform (right) and representative 2D class averages (middle left), and scarce bottom views belonging to channels with closed RCK rings. Representative particles are highlighted with red circles. **b**. Flow chart for data processing and the final maps of RCK gating rings (Methods). **c**. Gold-standard Fourier shell correlation (GSFSC) curve for the final RCK open conformation map shown in the red box in b. **d**. Angular distribution of particles used in the final reconstruction of the RCK open conformation map. **e**. Gold-standard Fourier shell correlation (GSFSC) curve for the final RCK intermediate conformation map shown in the blue box in b. **f**. Angular distribution of particles used in the final reconstruction of the RCK intermediate conformation map. **g**. Local resolution map of the RCK open conformation shown in two orientations. **h**. Local resolution map of the RCK intermediate conformation shown in two orientations.

**S5 Fig. Refinement of Kch TM domain. a.** Extra refinement steps performed on the Kch-2xRCK apo dataset aimed at resolving full-length Kch. **b.** Final reconstruction of full-length Kch. **c**. Angular distribution of particles used in the final reconstruction of the full-length Kch-2xRCK map. **d**. Gold-standard Fourier shell correlation (GSFSC) curve for the final map of full-length Kch-2xRCK. **e.** Kch TM3-6 model built inside the density map in a rainbow scheme depicting TM3 as cyan, and TM6 as red. **f.** Top and side views of Kch-2xRCK density map fitted with the tetrameric model of Kch TM3-6.

**S6 Fig. Open RCK Density Fits. a.** Individual secondary structures of the open RCK gating ring (sticks) and their densities (grey mesh) divided by alpha helices or **b** beta sheets.

**S7 Fig. Intermediate RCK Conformation Density Fits. a.** Individual secondary structures of the intermediate RCK gating ring (sticks) and their densities (grey mesh) divided by alpha helices or **b** beta sheets.

**S8 Fig. RCK Topology. a** Model of the open Kch RCK monomer colored in Rainbow from its N terminus (Deep Blue) to the C terminus (Red). **b** Topology diagram of the Kch RCK monomer.

**S9 Fig. Intermediate RCK ring interfaces. A** Cartoon depicting the arrangement of RCKs in the two-fold symmetric intermediate RCK ring with cylindrical models highlighting the different types of interfaces seen in the structure. Two RCK dimers are traced, an open one (yellow) and a closed one (brown).

**S10 Fig. Cryo-EM data processing of the closed RCK ring and open RCK ring with zinc from Kch** **WT**. **a**. A representative micrograph of Kch WT (Left), its Fourier transform (right) and representative 2D class averages (middle). Representative particles are highlighted with red circles. **b**. Flow chart for data processing and the final maps of the closed RCK gating ring (Methods). **c**. Gold-standard Fourier shell correlation (GSFSC) curve for the final RCK closed conformation map shown in b (left). **d**. Angular distribution of particles used in the final reconstruction of the RCK closed conformation map. **e.** Local resolution map of the RCK closed conformation shown in two orientations. **f.** Gold-standard Fourier shell correlation (GSFSC) curve for the final RCK open conformation map shown in b (right). **g.** Angular distribution of particles used in the final reconstruction of the RCK open conformation map. **h.** Local resolution map of the RCK open conformation shown in two orientations.

**S11 Fig.** **RCK Open Conformation in the Presence of Zinc Density fits. a.** Individual secondary structures of the closed RCK gating ring (sticks) and their densities (grey mesh) divided by alpha helices or **b** beta sheets.

**S12 Fig. RCK Closed Conformation Density Fits. a.** Individual secondary structures of the closed RCK gating ring (sticks) and their densities (grey mesh) divided by alpha helices or **b** beta sheets.

**S13 Fig. RCK polymers and “tail-to-tail” dimers. a-d.** Micrographs of marked samples of Kch, with representative 2D classification of circled particles present below or beside the micrograph. **e.** Density map of the two RCK rings in a “tail-to-tail” dimer present in the WT Kch sample purified with a membrane preparation step. **f.** Cutout zoomed in view of the interface between the two RCK gating rings that interact within the “tail-to-tail” dimer. **g.** Size exclusion profiles of Kch WT and H241A mutant showing a significant decrease in free RCK expression caused by the mutation.

**S14 Fig. RCK dimer & tetramer interface hinge movements. a.** Two orientations of the dimerization interface where RCK1 (yellow) has been aligned between the open (red) and closed (magenta) conformations, highlighting the movement seen on RCK2 in the other side of the dimerization interfaced interface. **b.** Model depiction of the RCK tetramerization interface where helices 4 & 5 of RCK1 from dimer 1 (green) and helices 4 & 5 of RCK2 from dimer 2 (blue) are compared between the open and **c** closed conformations in two orientations. **d.** Three orientations of the tetramerization interface where RCK1 (green) has been aligned between the open (blue) and closed (magenta) interfaces, highlighting the movement seen on RCK2 from dimer 2.

**S15 Fig. RCK dimer comparison with previous. a,** Model depiction of the RCK dimer in the open conformation (cyan) structurally aligned to the crystal structure obtained by Y. Jiang et al (black). **b.** Model depiction of the RCK dimer in the closed conformation (magenta) structurally aligned to the crystal structure obtained by Y. Jiang et al (black) in two orientations **c.** Alignment of the crystal structure obtained by Y. Jiang et al (black) with the open (cyan) and closed (magenta) RCK dimers.

**S16 Fig. Function of Kch mutants. a-i.** ACMA fluorescence quench traces of the indicated mutants in the absence (black) or presence of 30 μM Zn^2+^ (red). Empty and Kch WT controls are shown in dashed traces in different shades of gray. **j.** Model depiction of an RCK monomer with mutation sites marked.

**S17 Fig. Fibers assembled from decameric RCK rings. a.** A representative micrograph of the soluble RCK fraction purified from the full-length Kch, its Fourier transform (right) and representative 2D class averages (middle). Representative particles are highlighted with red circles. **b**. Flow chart for data processing and the final maps of the closed RCK gating ring (Methods). **c**. Gold-standard Fourier shell correlation (GSFSC) curve for the final RCK fiber map shown in b (left). **d**. Angular distribution of particles used in the final reconstruction of the RCK gating rings within the fiber density map. **e.** Two decameric RCK ring models built within the RCK fiber density map, cutout on the right highlighting the interface between two rings stacked in the fiber.

**Supplementary Table 1. Summary of cryo-EM data collection, processing, and structural refinement**

|  | Apo RCK gating ring in open conformation (EMD-72842, PDB 9YE0) | Apo RCK Gating ring in intermediate conformation (EMD-72908, PDB 9YFV) | RCK Gating ring in closed conformation (EMD-72932, PDB 9YGL) | RCK Gating ring in open conformation + Zn^+2^ (EMD-73056, PDB 9YKQ) |
| --- | --- | --- | --- | --- |
| **Data collection and processing** |  |  |  |  |
| Magnification | 105,000 | 105,000 | 105,000 | 105,000 |
| Voltage (kV) | 300 | 300 | 300 | 300 |
| Electron exposure (e–/Å^2^) | 50 | 50 | 50 | 50 |
| Defocus range (μm) | [-2.0, -0.8] | [-2.0, -0.8] | [-2.0, -0.8] | [-2.0, -0.8] |
| Pixel size (Å) | 0.832 | 0.832 | 0.832 | 0.832 |
| Symmetry imposed | C4 | C2 | C4 | C4 |
| Initial particle images (no.) | 18,170,630 | 18,170,630 | 1,560,525 | 1,560,525 |
| Final particle images (no.) | 197,637 | 192,194 | 55,071 | 185,079 |
| Map resolution (Å)  FSC threshold | 2.9 | 2.9 | 2.9 | 3.2 |
|  | 0.143 | 0.143 | 0.143 | 0.143 |
| Map resolution range (Å) | 2.6-4.0 | 2.6-4.0 | 2.5-4.0 | 2.65-4.4 |
|  |  |  |  |  |
| **Refinement** |  |  |  |  |
| Initial model used (PDB code) | 1ID1 | 1ID1, Alphafold2 (Colab Multimer Prediction) | Alphafold2 (Colab Multimer Prediction) | 1ID1 |
| Model resolution (Å)  FSC threshold | 3 | 3 | 3.1 | 3.3 |
|  | 0.5 | 0.5 | 0.5 | 0.5 |
| Model resolution range (Å) | 2.8-3.0 | 2.9-3.1 | 2.8-3.1 | 3.1-3.3 |
| Map sharpening *B* factor (Å^2^) | 143.5 | 144.1 | 108.9 | 197.2 |
| Model composition  Non-hydrogen atoms  Protein residues  Ligands | 9048 | 9076 | 9272 | 9032 |
|  | 1224 | 1216 | 1232 | 1224 |
|  | 0 | 0 | 0 | 0 |
| *B* factors (Å^2^)  Protein  Ligand | 042.3 | 41.1 | 55.2 | 74.3 |
|  | 0 | 0 | 0 | 0 |
| R.m.s. deviations  Bond lengths (Å)  Bond angles (°) | 0.002 | 0.002 | 0.002 | 0.002 |
|  | 0.454 | 0.52 | 0.447 | 0.423 |
| Validation  MolProbity score  Clashscore  Poor rotamers (%) | 1.4 | 1.55 | 1.67 | 1.43 |
|  | 4.52 | 5.69 | 6.83 | 5.15 |
|  | 1.68 | 3.12 | 2.43 | 1.59 |
| Ramachandran plot  Favored (%)  Allowed (%)  Disallowed (%) | 98.76 | 96.33 | 99.34 | 98.59 |
|  | 1.24 | 6.67 | 0.66 | 1.41 |
|  | 0 | 0 | 0 | 0 |

**Supplementary Table 2. Sequence of primers**

/
